# The Murid Herpesvirus-4 gL Regulates an Entry-Associated Conformation Change in gH

**DOI:** 10.1371/journal.pone.0002811

**Published:** 2008-07-30

**Authors:** Laurent Gillet, Susanna Colaco, Philip G. Stevenson

**Affiliations:** Division of Virology, Department of Pathology, University of Cambridge, Cambridge, United Kingdom; University of California San Francisco, United States of America

## Abstract

The glycoprotein H (gH)/gL heterodimer is crucial for herpesvirus membrane fusion. Yet how it functions is not well understood. The Murid Herpesvirus-4 gH, like that of other herpesviruses, adopts its normal virion conformation by associating with gL. However, gH switched back to a gL-independent conformation after virion endocytosis. This switch coincided with a conformation switch in gB and with capsid release. Virions lacking gL constitutively expressed the down-stream form of gH, prematurely switched gB to its down-stream form, and showed premature capsid release with poor infectivity. These data argue that gL plays a key role in regulating a gH and gB functional switch from cell binding to membrane fusion.

## Introduction

Enveloped viruses deliver their capsids to the cytoplasm by fusing with either the plasma membrane or the limiting membranes of endocytic vesicles. Many viruses use a single glycoprotein for both binding and membrane fusion. Herpesviruses, by contrast, use at least four [1], [2]; their entry mechanisms are implicitly more complex. Proteins specific to individual herpesviruses can modulate fusion, for example the Herpes simplex virus gD brings together gB and gH/gL after ligand engagement to trigger fusion [3], [4], and the Epstein-Barr virus gp42 binds gH/gL to promote B cell over epithelial cell fusion [5]. But the core fusion machinery of gB, gH and gL is conserved. Down-stream fusion events are therefore likely to be common to all herpesviruses.

We have analyzed gB and gH/gL using the MHV-68 isolate of Murid Herpesvirus-4 (MuHV-4) [6]–[8]. This gamma-herpesvirus has subtle differences from the more extensively studied Herpes simplex virus, for example it lacks an obvious homolog of gD and fuses after endocytosis rather than at the plasma membrane [9]. Nevertheless, the conservation of gB and gH/gL implies that they operate similarly in these and other herpesviruses once the fusion mechanism is engaged. Incoming MuHV-4 virions first bind to cell surface heparan sulphate proteoglycans via gH/gL or gp70 [10], [11]. Gp150 displacement then allows further, heparan sulphate-independent binding, probably via gB [10], [12]. Virions are endocytosed and transported to LAMP-1^+^ late endosomes [13]; membrane fusion then releases virion capsids into the cytoplasm; and these move to the nuclear margin for genome delivery [13].

Both gB and gH have hydrophobic loops that could participate directly in fusion [14], [15]. Structural analysis of the Herpes simplex virus gB has [14] shown how these might be made available. Comparison with the Vesicular stomatitis virus glycoprotein G [16] suggests that the crystallized form of gB, with its distinct crown formed mainly from C-terminal domains, is a down-stream form. Consistent with this, the Murid Herpesvirus-4 (MuHV-4) gB switches during entry from having readily accessible N-terminal epitopes and poorly accessible epitopes involving C-terminal domains to the converse [13]. The N-terminal domains are involved in cell binding [10]. Thus, gB on extracellular MuHV-4 virions adopts a conformation that allows cell-binding, then following endocytosis undergoes a pH-dependent conformation switch that leads to membrane fusion. Whether this conformation change itself drives membrane fusion, as for Vesicular stomatitis virus, or whether further conformation changes are required along the lines suggested by Heldwein et al. [14], remains unclear.

While quite a lot is known about gB, much less is known about gH/gL. Understanding gH/gL therefore presents the major obstacle to understanding herpesvirus entry. Unlike gH, gL lacks obvious fusion loops or a trans-membrane domain. Its contribution to membrane fusion is therefore likely to be indirect. So far, gL has been defined only as a chaperone for gH [9], [17], [18]. Some herpesviruses need gL to get gH into virions [17], [18]; Pseudorabiesvirus does not, but remains non-infectious without gL [19]; MuHV-4 lacking gL both incorporates gH into virions and remains infectious [20]. These differences may reflect how gB and gH associate. The Herpes simplex virus gB and gH come together only when gD is ligated [3], [4], so their interaction is likely to involve extracellular domains; for gH, this would imply a requirement for gL. The MuHV-4 gB and gH associate constitutively and independently of gL, probably via an intra-membrane interaction [21]. Because MuHV-4 does not require gH/gL for viability, it provides a convenient tool with which to explore gL-dependent down-stream events in viral entry.

gL^−^ MuHV-4 virions do show some attenuation relative to the wild-type. One deficit is reduced binding to BHK-21 fibroblasts [20]. This reflects that gH/gL binds to GAGs, whereas gH alone does not [11]. However, gL-deficient virions infect NMuMG epithelial poorly even though they bind to them quite well [20]. gL must therefore have another function that is important for NMuMG cell infection. Here we identify an entry-associated conformation change in gH that was consistent with gL dissociation in late endosomes, the site of MuHV-4 capsid release. When virions lacked gL, gH and gB both adopted their down-stream forms prematurely. This led to premature capsid release and poor infectivity. An important function of gL is therefore to regulate the gB and gH conformation changes that lead to viral membrane fusion.

## Results

### The MuHV-4 gH changes conformation after endocytosis

**Figure 1:**
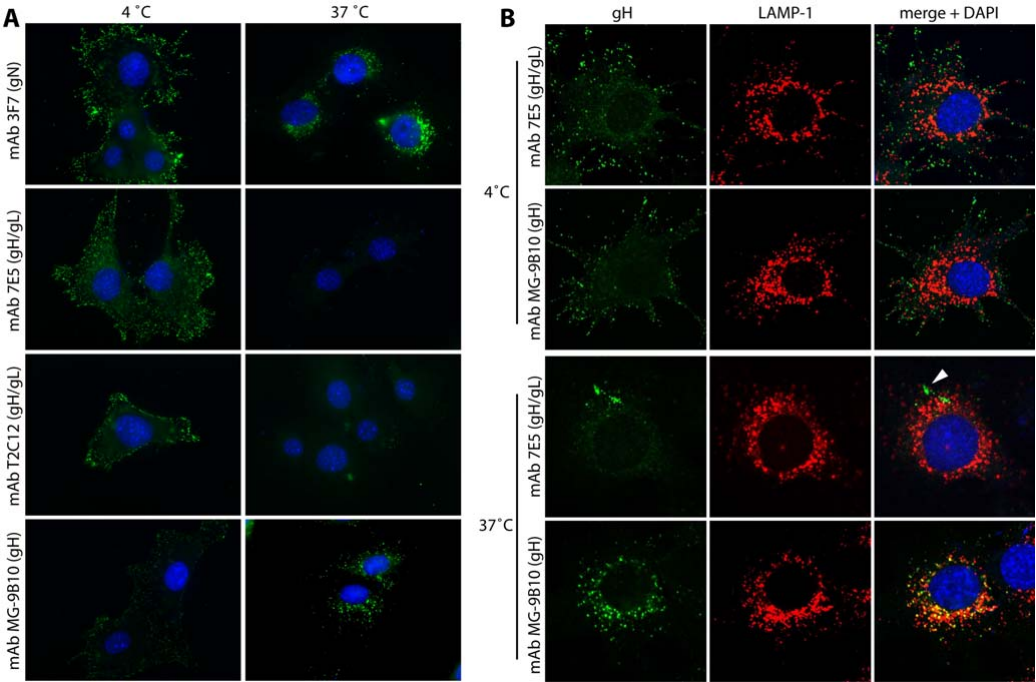
Glycoprotein H changes its antigenicity after endocytosis. A. NMuMG cells were exposed to MuHV-4 virions (3 p.f.u./cell, 2 h, 4°C), washed ×3 with PBS and either fixed immediately (4°C) or after a further 2 h incubation at 37°C (37°C). The cells were then stained for gH (MG-9B10), gH/gL (T2C12, 7E5) or gN (3F7) as an invariant control. Glycoprotein staining is green; nuclei are counterstained with DAPI (blue). The data are representative of 5 experiments, and similar results were obtained with 3 other gH-specific/gH/gL-specific mAb pairs. Single cells are shown for optimal resolution. In this as in subsequent figures, each individual cell is fully representative of at least 75% of the total examined (n>100). B. Cells were infected as in A, then stained for LAMP-1 (red) plus either gH (mAb MG-9B10) or gH/gL (mAb 7E5) (green). Co-localization is yellow. The arrow indicates residual gH/gL staining that does not colocalize with LAMP-1.

MuHV-4 infects adherent cells via endocytosis. Capsid release is pH-dependent and occurs when virions reach the late endosomes/lysosomes marked by lysosome-associated membrane protein-1 (LAMP-1) [13]. Capsid release is associated with a conformation change in gB. To test whether gH also changes, we examined its antigenicity before and after endocytosis (Fig. 1) using mAbs that distinguish gL-dependent and gL-independent gH conformations [9], [20]. The gH on wild-type virions is mostly bound to gL [20]. MAbs requiring both gH and gL for recognition, such as 7E5 and T2C12, accordingly stained incoming virions strongly at the cell surface (Fig. 1A). However, this staining was lost after endocytosis. In contrast, the gH-only-specific mAb MG-9B10 [20] gave weak staining at the cell surface and much stronger staining after endocytosis. Other gH-only-specific mAbs such as MG-1A2 and MG-2E6 [20] gave similar results to MG-9B10. The corresponding gH and gH/gL epitopes are all conformational, as the mAbs do not recognize denatured virions, and can all be expressed in the absence of other virion proteins on transfected cells. Therefore gH did not appear to be denatured or disguised, but rather switched from a gL-dependent to a gL-independent conformation.

### The MuHV-4 gH changes conformation in LAMP-1^+^ endosomes

The disappearance of gH/gL epitopes coincided with virions reaching LAMP-1^+^ late endosomes; any residual gH/gL-specific staining after incubation at 37°C was confined to LAMP-1^−^ compartments (Fig. 1B, arrow). In contrast, gH-only staining co-localized with LAMP-1. Most virion gH therefore switched from gH/gL on extracellular virions to gH-only in late endosomes. Thus, the gB conformation switch, the gH conformation switch and capsid release all coincided in time and place.

### The gH/gL conformation changes are pH-dependent

**Figure 2:**
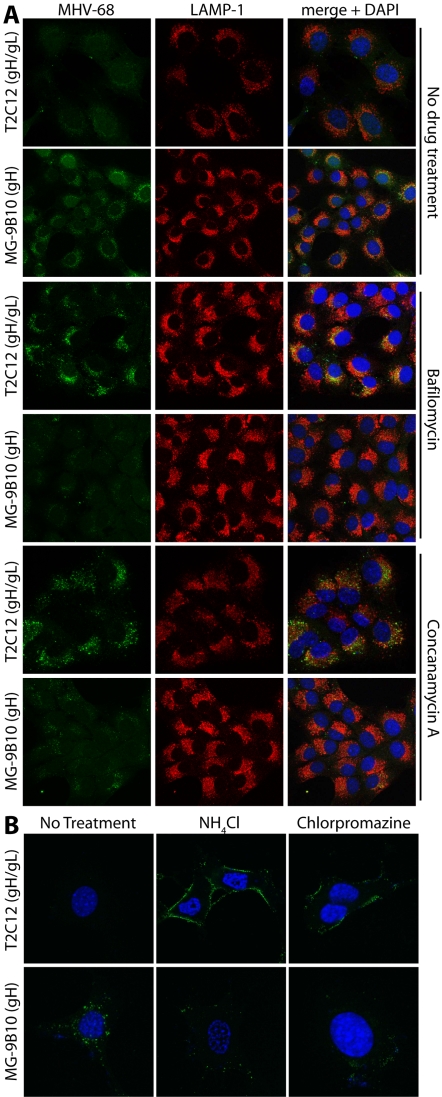
The gH/gL conformation change is pH-dependent. A. MuHV-4 virions were bound to NMuMG cells (2 h, 4°C) with or without bafilomycin (500 nM) or concanamycin A (1 µM). The cells were then washed in PBS to remove unbound virions, further incubated with or without drugs (2 h, 37°C), fixed, permeabilized and stained for gH or gH/gL plus LAMP-1. Nuclei were counterstained with DAPI. B. In a similar experiment, MuHV-4 virions were bound to NMuMG cells (2 h, 4°C) with or without NH_4_Cl (100 mM) or chlorpromazine (3 µg/ml). Unbound virus was removed by PBS wash and the cells incubated further (37°C, 2 h), again with or without drug treatment, before fixing, permeabilizing and staining for gH or gH/gL. Although NH_4_Cl is generally considered to block endosomal acidification, here it also seemed to block virion endocytosis.

The localization of the gH conformation switch to LAMP-1^+^ late endosomes suggested that it required low pH. This was confirmed with inhibitors of lysosomal acidification (Fig. 2A). After incubating virions with cells at 37°C without drugs, gH-only staining predominated over gH/gL. But when the cells were treated with bafilomycin or Concanamycin A, although virions were still endocytosed and transported to LAMP-1^+^ endosomes, gH/gL staining was preserved and gH-only staining correspondingly reduced. In particular, gH/gL staining now co-localized with LAMP-1, something never observed without drug treatment.

Blocking endocytosis with chlorpromazine also blocked the gH conformation change, as did ammonium chloride treatment (Fig. 2B). Although the latter classically blocks endosomal acidification, it also blocked MuHV-4 endocytosis. Such an effect has been observed before [22]. Thus, in contrast to bafilomycin and Concanamycin A, the effect of NH_4_Cl could not be attributed to a rise in endosomal pH. However, it seemed clear from the bafilomycin and Concanamycin A treatments that the MuHV-4 gH conformation switch required virion delivery to low pH endosomes rather than just endocytosis.

### A lack of gL causes a post-binding entry deficit

**Figure 3:**
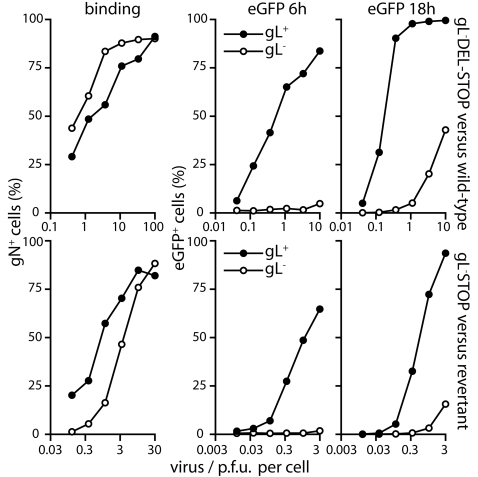
gL^−^ virions show a post-binding deficit in NMuMG cell infection. NMuMG cells were exposed to gL^+^ or gL^−^ MuHV-4 virions (3 h, 37°C), then washed ×3 in PBS. Equivalent samples were then either fixed, permeabilized and stained for gN with mAb 3F7 to determine virion uptake by flow cytometry; or incubated for a further 3 h or 15 h, after which viral eGFP expression was assayed by flow cytometry of intact cells. Each point shows the result for 10,000 cells. Equivalent results - the infectivity of gL^−^ viruses was <1% that of gL^+^ viruses for an equivalent level of cell binding - were obtained in a repeat experiment.

The apparent dissociation of gL from gH during MuHV-4 entry suggested that the gH-only conformation is functionally important, and therefore that gL has a more complicated role than merely folding gH for cell binding. One prediction of this would be that removing gL has consequences for post-binding entry events. We looked for evidence of a gL-dependent post-binding infection deficit by binding gL^+^ and gL^−^ virions to NMuMG cells for 3 h, washing off any unbound virions, and then comparing the initial virion uptake with subsequent viral eGFP expression (Fig. 3). We compared first a gL^−^ mutant with the wild-type, and then an independent gL^−^ mutant with its revertant. Cell binding was similar between the gL^−^ and gL^+^ viruses, but virus-driven eGFP expression was markedly different. After 6 h, eGFP expression by the gL^−^ viruses was virtually undetectable: for an equivalent level of binding, the infectivity of gL^+^ MuHV-4 for NMuMG cells at 6 h was at least 100 times greater than that of gL^−^ mutants. Even after 18 h, gL^+^ viruses showed at least 10-fold more eGFP expression for an equivalent level of binding. gL^−^ virions therefore showed a marked post-binding infection deficit in NMuMG cells.

### gL^−^ virions show premature capsid release

**Figure 4:**
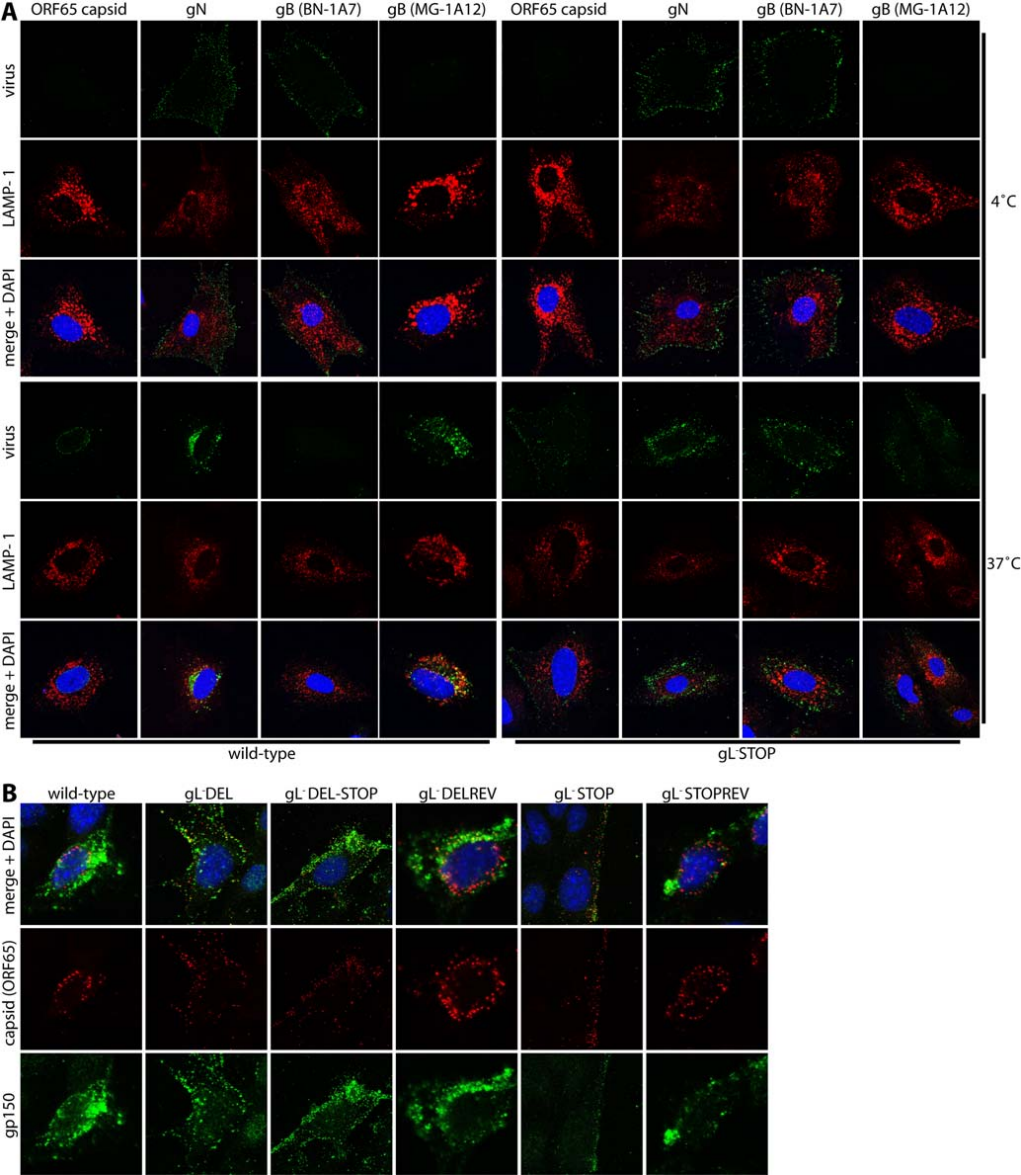
gL^−^ virions show altered glycoprotein and capsid distributions and gB conformation changes. A. NMuMG cells were exposed to wild-type or gL^−^ virions (2 h, 4°C, 3 p.f.u./cell) then washed ×3 in PBS and either fixed immediately (4°C) or first incubated (2 h, 37°C) to allow virion endocytosis (37°C). The cells were then permeabilized and stained for LAMP-1 plus MuHV-4 capsid or glycoprotein antigens as shown. Nuclei were counterstained with DAPI. Colocalization appears as yellow in the merged images. Equivalent data were obtained in 3 further experiments. B. MCCD epithelial cells were incubated with a range of different gL^−^ or gL^+^ viruses (2 h, 4°C, 3 p.f.u./cell), then washed ×3 with PBS and incubated further (2 h, 37°C) to allow virion endocytosis. The cells were then stained for gp150, an abundant virion glycoprotein, with mAb LS-B11 (IgG1) and for ORF65 capsid with mAb MG-12B8 (IgG2a). Nuclei were counterstained with DAPI.

We tracked the entry of gL^−^ virions into NMuMG cells by immunofluorescence (Fig. 4A). MAb MG-12B8 recognizes an ORF65 capsid epitope that is inaccessible on virions until they have uncoated [23]. There was accordingly no MG-12B8 staining of either wild-type or gL^−^ virions after binding to NMuMG cells at 4°C (Fig. 4A). After incubation at 37°C, the capsids of wild-type virions had become visible and reached the nuclear margin. In contrast, gL^−^ virion capsids, although accessible, remained scattered throughout the cytoplasm. The same abnormal capsid distribution after incubation at 37°C was observed for a range of different gL mutants, but not for their revertants or the wild-type (Fig. 4B). Since capsids are normally released only from perinuclear late endosomes, the gL^−^ virions must have released their capsids prematurely in the entry pathway.

**Figure 5:**
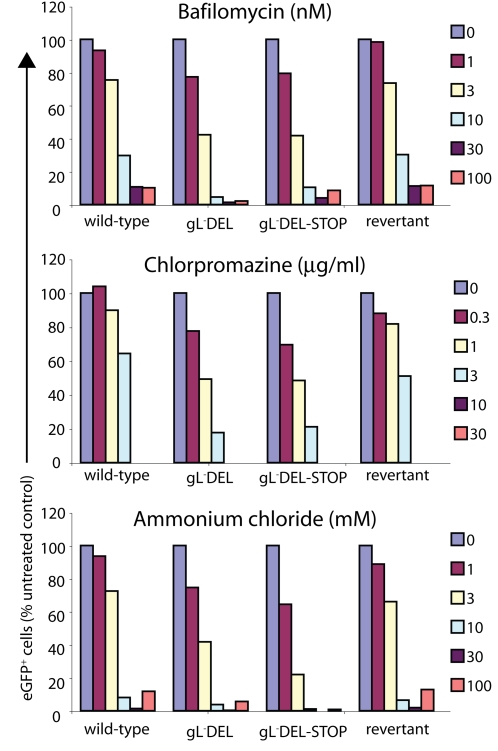
gL-deficient MuHV-4 remains dependent on endocytosis and lysosomal acidification. BHK-21 cells were treated as indicated for 2 h, then infected with gL- (gL^−^DEL, gL^−^DEL-STOP) or gL^+^ (wild-type, revertant) MuHV-4 (1 p.f.u./cell). Drug treatment was maintained throughout the course of infection. Infection rates were determined by flow cytometric assay of viral eGFP expression after 18 h. Each point shows the result for 10,000 cells. Equivalent results were obtained in a repeat experiment.

The glycoproteins of gL^−^ virions were still internalized rather than incorporated into the plasma membrane. Surprisingly, infection by gL^−^ virions also remained at least as sensitive to inhibitors of endocytosis and lysosomal acidification as that of the wild-type or gL^+^ revertants (Fig. 5). This may reflect that low pH has a wider importance for MuHV-4 entry than switching the conformation of virion glycoproteins. For example, it could be required to reveal a key cellular ligand. Simply exposing cell-bound virions to low pH triggered the gH conformation change at best poorly (data not shown). We have observed this also for gB [13]. Therefore the MuHV-4 gH and gB conformation switches were not simply pH-dependent as for Vesicular stomatitis virus glycoprotein G [16]: other features of the endosome environment were required. Nevertheless, low pH was essential for efficient gH and gB switching and for capsid release.

It is important to note that most gL^−^ virions were non-infectious (Fig. 3). Therefore most of the prematurely capsids of gL^−^ virions were destined not to reach nuclear pores. What gL^−^ infection there was may have depended on a few of these capsids being transported, or on some virions still managing to follow the normal pathway to late endosomes without premature membrane fusion.

### gL^−^ virions show gB conformational instability

**Figure 6:**
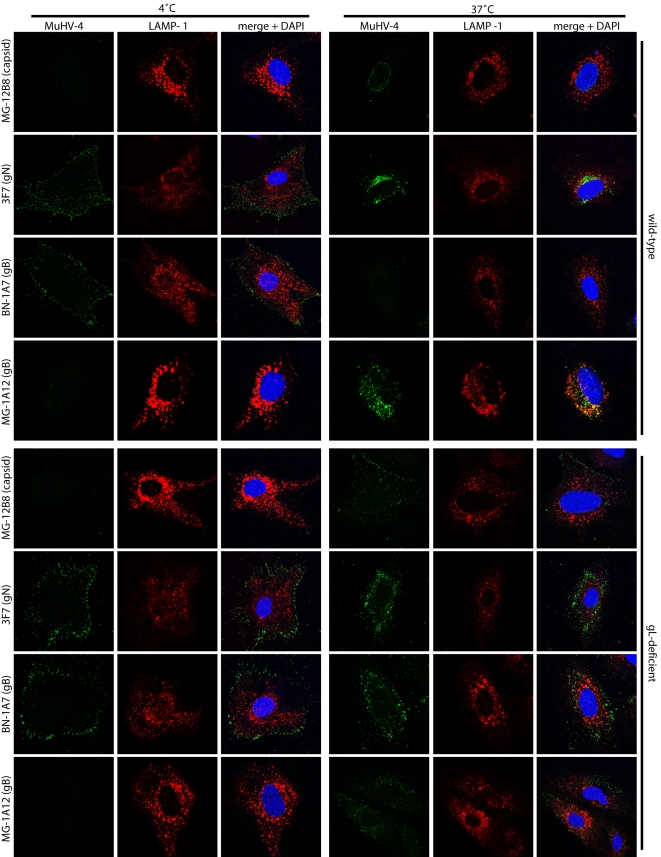
gL-deficient virions show abnormal entry in NIH-3T3 fibroblasts. gL^+^ and gL^−^ virions were bound to adherent NIH-3T3 cells (2 h, 4°C). Unbound virions were then removed by PBS wash. The cells were then either fixed (4°C) or first incubated (2 h, 37°C) to allow virion endocytosis and then fixed (37°C). All cells were then permeabilized and stained for MuHV-4 virion components (green) and LAMP-1 (red) as shown. Nuclei were counter-stained with DAPI (blue). In the absence of gL, both capsids and glycoproteins remained peripheral. gB conformation changes were also affected. Notably, MG-1A12^+^ gB appeared in peripheral, LAMP-1^−^ endosomes. Representative cells are shown.

Since MuHV-4 membrane fusion, as defined by capsid release, is associated with a conformation change in gB [13], we further analyzed incoming gL^−^ virions for changes in gB antigenicity. The gB of wild-type virions changed from BN-1A7^+^MG-1A12^−^ (pre-fusion) at the cell surface to BN-1A7^−^MG-1A12^+^ (post-fusion) in late endosomes (Fig. 4A). gL^−^ virions attached to the plasma membrane were also BN-1A7^+^MG-1A12^−^. However, unlike the wild-type, some acquired the MG-1A12 epitope before reaching LAMP-1^+^ endosomes. A premature gB switch to MG-1A12^+^ was also observed for gL^−^ virions in NIH-3T3 cells (Fig. 6). The gL-dependent conformational instability of gB was not marked, as considerable BN-1A7 staining remained outside LAMP-1^+^ endosomes. Nevertheless, as judged by capsid release this degree of instability was functionally important.

**Figure 7:**
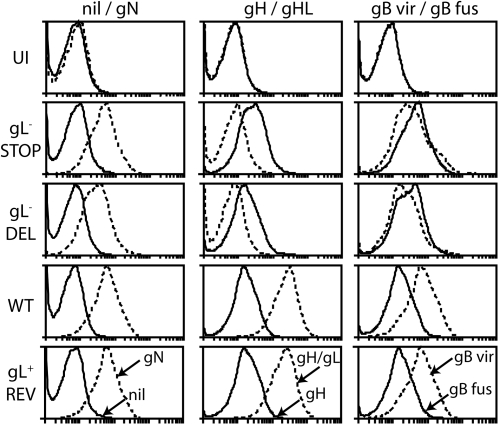
gL-dependent gB conformational instability in MuHV-4-infected BHK-21 cells. BHK-21 cells were left uninfected (UI) or infected with gL^−^ (gL^−^STOP, gL^−^DEL) or gL^+^ (wild-type, gL^−^DEL revertant) viruses (2 p.f.u./cell, 18 h). The cells were then trypsinized and stained with secondary antibody only (nil, solid lines) or for gN (mAb 3F7, dotted lines), gH-only (mAb MG-9B10, solid lines), gH/gL (mAb T2C12, dotted lines), the mAb BN-1A7^+^ virion gB conformation (gB vir, dotted lines) or the mAb MG-1A12^+^ pro-fusion gB conformation (gB fus, solid lines). The data are from 1 of 3 equivalent experiments.

Flow cytometry of infected BHK-21 cells provided further evidence of gB conformational instability in the absence of gL (Fig. 7). For an equivalent level of gN expression, BN-1A7 gB staining (pre-fusion) was weaker on cells infected with gL knockout viruses than with wild-type or revertant viruses, and MG-1A12 staining (post-fusion) was stronger. Thus, the premature capsid release of gL^−^ virions reflected gH being in its down-stream gH-only form from the start, and gB engaging prematurely in membrane fusion after endocytosis.

### gL deficiency compromises host entry

A key point with MuHV-4 is that *in vitro* molecular deficits can be linked to *in vivo* host colonization phenotypes. We have shown before that MuHV-4 gL knockouts colonize mice remarkably normally after intranasal inoculation of 10^4^ plaque-forming units [20]. However, the *in vitro* entry deficit of gL^−^ virions (Fig. 3) suggested that initial host entry might be impaired. Since host colonization by MuHV-4 is relatively dose-independent [24], such a deficit might not be apparent except at very low inoculation doses. We therefore compared gL^−^ (2 independent mutants) and gL^+^ (revertant and wild-type) viruses for their capacity to establish infection after intranasal inoculation of 1–100 p.f.u. (Table 1). gL disruption reduced significantly the rate of infection after low dose inoculation. Thus, while gL deficiency has little impact on MuHV-4 intra-host spread, it compromised severely the efficiency with which cell-free virions first entered naive hosts.

**Table 1:** Low dose *in vivo* infection by gL^+^ and gL^−^ MuHV-4.

	infecting dose		
virus	1 p.f.u.	10 p.f.u.	100 p.f.u.
wild-type	6/12^1^	12/12	12/12
gL^−^DEL	1/15	7/12	10/12
gL^−^STOP	2/15	7/12	10/12
revertant	4/12	12/12	12/12

1 infected mice/total exposed.

Mice were infected significantly better by wild-type MuHV-4 than by either the gL^−^DEL or gL^−^STOP mutants at 10 p.f.u./mouse (p<0.02 by Fisher's exact test) and 1 p.f.u./mouse (p<0.05). The revertant was not significantly different to wild-type. Comparison between 1 p.f.u. and 10 p.f.u. suggested that the *in vivo* infectivity of gL knockout virions is about 1/10 that of the wild-type.

## Discussion

Sheer complexity has made herpesvirus membrane fusion hard to unravel, for example Herpes simplex virus can undergo endosomal or plasma membrane fusion in different cell types [25]. However, the underlying molecular events are likely to be similar. Endocytic entry is easier to dissect because binding and fusion remain anatomically distinct. MuHV-4 capsid release after endocytic entry was associated with conformation switches in both gB and gH. Low pH was a key trigger of these switches and gL was a key negative regulator. Thus, by associating with newly synthesized gH in the endoplasmic reticulum gL sets virions for cell binding, and by dissociating again in late endosomes of the next cell it triggers membrane fusion.

Analogy with the Vesicular stomatitis virus glycoprotein G [15], [16] would suggest that the conformation changes in the MuHV-4 gB are intrinsic to its fusion mechanism, with the stable BN-1A7^+^ or MG-1A12^+^ forms representing the respective pre- and post-fusion states. This general model does not apply so easily to gH (gH/gL being pre-fusion and gH-only post-fusion), as gL^−^ virions fail to express gH/gL yet fuse more readily than the wild-type rather than less. It is possible that gL^−^ virions express another, as yet unidentified, pre-fusion form of gH. However, we have found no antigenic evidence of other forms (data not shown). Our working hypothesis is therefore that gH/gL and gH-only are both pre-fusion - gH/gL being up-stream of gH-only - and that further gH conformation changes accompany fusion itself. In support of this, gH-only epitopes tended to disappear after a 4 h 37°C incubation, whereas MG-1A12-type gB epitopes were maintained (data not shown). Thus, gL dissociation from gH appears to prime gH for membrane fusion rather than being a part of the fusion reaction per se.

A pre-fusion conformation change cannot be ruled out for gB either. Heldwein et al. [15] proposed that their gB conformation - which is probably a down-stream form [26] - undergoes further changes to reveal fusion loops. The neutralization epitopes mapped onto this structure [15] would also be difficult to explain if it were simply a post-fusion form. Thus, although the default explanation has to be that gB functions like the Vesicular stomatitis virus glycoprotein G because of their structural homology, it is also possible that the herpesvirus membrane fusion mechanism is different. gB and gH conformation changes must drive fusion, but this does not necessarily mean that every conformation change is part of the fusion mechanism. It is possible that the conformation changes described for Vesicular stomatits virus have for herpesviruses acquired a different function. But whatever the precise fusion mechanism of herpesviruses, a central role for the MuHV-4 gL in stabilizing gH and gB against engaging in fusion seemed clear.

The influence of gL on the conformation of gB was presumably indirect. A loss of gL would change gH/gL to gH-only and would therefore disrupt the interaction [27] between gH/gL and the gB N-terminus (gB-NT). This would not separate gH from gB, since their association is independent of both gL [21] and gB-NT [27], but could promote a conformation change in the gB extracellular domain. Consistent with such a scheme, MuHV-4 lacking gB-NT shows a gB conformational instability much like that of gL^−^ mutants [27]. The gB-NT-gH/gL interaction does not appear to play a significant role in stabilizing gH/gL, since gB-NT-deficient mutants maintain gH/gL expression and infect normally [27]. Therefore the gB conformation change alone is not sufficient for fusion: gL rather than gB-NT is the key fusion regulator. We envisage that the late endosomal milieu disrupts the gH/gL interaction, with low pH playing an important permissive role; gH then changes its conformation to allow hemi-fusion [28] and displace gB-NT; this then allows gB to switch to its pro-fusion form and complete the fusion reaction. In this way, the whole of membrane fusion can be regulated by gL. The neatness of this mechanism for regulating virion entry suggests that it will prove common to many herpesviruses.

## Materials and Methods

### Cells, viruses, mice

BHK-21 fibroblasts, NIH-3T3 fibroblasts, NMuMG epithelial cells, and MCCD epithelial cells were propagated as described [27]. Wild-type and gL^−^ viruses were derived from a cloned MuHV-4 BAC [29] and grown in BHK-21 cells [29]. Female BALB/c mice (Harlan, Bicester, U.K.), were infected intranasally with MuHV-4 under general anaesthesia when 6–8 weeks old, in accordance with the animal care guidelines of Home Office Project Licence 80/1992 [30]. For virus titrations, lungs were removed post-mortem, freeze-thawed, and homogenized in 1 ml PBS. Serial dilutions were then plated onto BHK-21 cell monolayers. These were fixed with 4% formaldehyde after 4 days and stained with 0.1% toluidine blue for plaque counting [30].

### Immunofluorescence

Cells on glass coverslips [31] were exposed to MuHV-4 virions (3 p.f.u./cell) to allow binding, in most experiments at 4°C. The cells were then washed ×3 in PBS to remove unbound virions and shifted to 37°C to allow endocytosis. They were fixed in 4% paraformaldehyde (30 min) either before or after endocytosis, then permeabilized with 0.1% Triton X-100. Where indicated, bafilomycin, concanamycin A, NH_4_Cl or chlorpromazine was added from 2 h pre-infection to the time of fixation. Viral glycoproteins were detected with murine mAbs (Table 2) at 1–10 µg/ml plus Alexa488- or Alexa568-labeled anti-mouse IgG (Invitrogen, Paisley, U.K.) or Alexa488- or Alexa633- labeled anti-mouse IgG_1_ plus Alexa568- labeled anti-mouse IgG_2a_ at 1 µg/ml. LAMP-1 was detected with mAb 104B (BD-Pharmingen, Oxford, U.K.) plus Alexa488- or Alexa568- labeled anti-rat IgG (Invitrogen). Nuclei were counterstained with DAPI. Fluorescence was visualized on a Leica SP2 confocal microscope.

**Table 2:** MAbs used in this study.

MAb	Target	Isotype	Epitope^1^
BN-1A7	gB	IgG2a	conformational
MG-1A12	gB	IgG2a	conformational
LS-B11	gp150	IgG1	linear
7E5	gH/gL	IgG2a	conformational
T2C12	gH/gL	IgG2a	conformational
MG-9B10	gH	IgG2b	conformational
3F7	gN	IgG2a	linear
MG-12B8	ORF65 (capsid)	IgG2a	linear

1 As defined by recognition or not of denatured protein on immunoblots.

### Flow cytometry

BHK-21 cells were infected with gL^+^ or gL^−^ MuHV-4 (2–5 p.f.u./cell), then trypsinized and either analyzed directly for viral eGFP expression (FACSCalibur, BD Biosciences) or first stained (1 h, 4°C) with MuHV-4 glycoprotein-specific mAbs plus fluorescein-labeled anti-mouse IgG (Dako Cytomation) [32].
