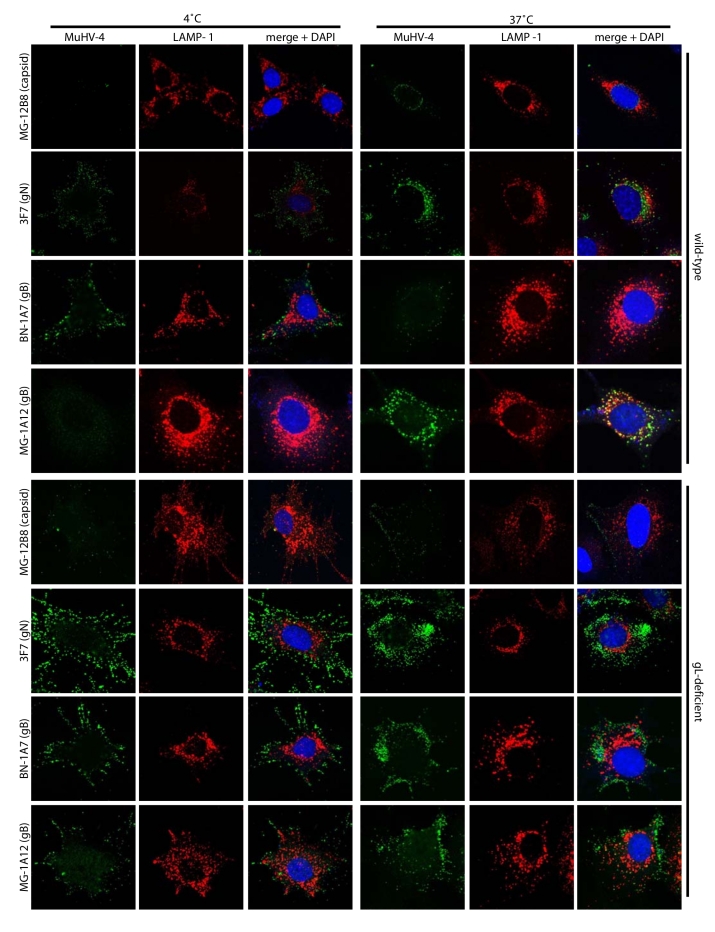# Correction: The Murid Herpesvirus-4 gL Regulates an Entry-Associated Conformation Change in gH

**DOI:** 10.1371/annotation/4c409091-3915-489b-ae61-48352c8e69b2

**Published:** 2008-09-05

**Authors:** Laurent Gillet, Susanna Colaco, Philip G. Stevenson

The image for Figure 6 represents the wrong data set. Please view the correct figure here:

**Figure:**